# Hematologic and Biochemical Reference Interval Development and the Effect of Age, Sex, Season, and Location on Hematologic Analyte Concentrations in Critically Endangered Yangtze Finless Porpoise (*Neophocaena asiaeorientalis* ssp. *asiaeorientalis*)

**DOI:** 10.3389/fphys.2019.00792

**Published:** 2019-07-11

**Authors:** Ghulam Nabi, Todd R. Robeck, Yujiang Hao, Ding Wang

**Affiliations:** ^1^Institute of Hydrobiology, Chinese Academy of Sciences, Wuhan, China; ^2^University of Chinese Academy of Sciences, Beijing, China; ^3^SeaWorld Parks & Entertainment, Orlando, FL, United States

**Keywords:** clinicopathological, critically endangered, Poyang Lake, reference interval, Yangtze finless porpoise

## Abstract

In this study, references intervals for 49 clinicopathological parameters were established for the critically endangered Yangtze finless porpoise (YFP) (*Neophocaena phocaenoides asiaorientalis*). Both from the wild (Poyang Lake) and seminatural (Tian-E-Zhou Oxbow) populations, individual blood samples from 188 animals were collected from 2009 to 2017 and from 2002 to 2015, respectively. For reference interval determination, we used a non-parametric bootstrap-based procedure to determine the 95th percentiles and the associated 90% confidence interval for each analyte. Our results indicated a need to partition the analyte concentrations by sex, age group, or pregnancy; however, we did not find a need to partition results by location. We then used a linear mixed model to determine if evidence existed for mean differences between location with sex and season as covariates and age group as the clustered random variable on mean hematological parameters in the YFP. We found that 88% of the analytes were significantly different between locations. Within the covariates, sex and season showed 31 and 69% significant difference in mean distributions, respectively. Additionally, age group provided a significant source of variation in 25% of the analytes. In summary, our finding suggests that analytes should be grouped according to sex, age, and reproductive status (non-pregnant and non-lactating, pregnant and lactating). Furthermore, we have provided the first set of reference intervals for 49 clinicopathological parameters that could provide guidelines for the initial evaluation of individuals during health assessments.

## Introduction

Hematological and biochemical analyses are fundamental components of individual animal health assessment. Typically, the normality of each analyte concentration which has been determined through these analyses is assessed by comparing them against population-level reference intervals (Stockham and Scott, 2008). Further, sensitivity for the detection of physiologic abnormalities is improved when reference intervals have been developed which control for normal variation due to environmental, e.g., season, temperature, or physiological, e.g., sex, age, and reproductive state, effects (Monke et al., 1998; Bossart et al., 2001; Stockham and Scott, 2008). While reference interval development has been well established and relied upon within domestic and *ex situ* exotic animals and human medicine (Lewis et al., 1998; Monke et al., 1998; Venn-Watson et al., 2007; Latimer, 2011; Nollens et al., 2019), their establishment within wildlife populations has only recently gained traction. This is primarily due to the inaccessibility of animals and insufficient numbers of animals for proper reference interval development (Gales, 1992; Koopman et al., 1999; Clinical and Laboratory Standards Institute [CLSI], 2008). For wild cetacean species, species which are at risk toward ever increasing anthropogenic competition and encroachment, a relative recent push for *ex situ* cetacean population health assessments has resulted in a few species, primarily bottlenose dolphins and beluga whales, whereby initial attempts at establishing reference intervals have been made (St Aubin et al., 2001; Ruiz et al., 2009; Schwacke et al., 2009; Norman et al., 2012).

The Yangtze finless porpoise (YFP) (*Neophocaena asiaeorientalis* ssp. *asiaeorientalis*) is a critically endangered freshwater cetacean, endemic to the Yangtze River, Poyang Lake (PL), and Dongting Lake (DL) (Wang et al., 2013). Establishing hematological and biochemical reference intervals for wild YFP will provide essential baseline data from which the health of future populations can be evaluated (Perri et al., 2017). This information is critical due to the increasing anthropogenic threats in the form of toxic byproducts (Dong et al., 2006; Leeuw et al., 2009; Schelle, 2010; Wang et al., 2010) and acoustic pollutants (Smith and Reeves, 2000; Akamatsu et al., 2002; Schelle, 2010), to which these species are increasingly being exposed. Currently, there are about 450 YFPs in PL, 90 in DL, and the remaining estimated 505 in Yangtze River (Mei et al., 2014). In 1992, the Chinese Government identified Tian-E-Zhou Oxbow (TZO), a large lake in the middle region of Yangtze River, as a natural, protected *ex situ* reserve to translocate YFPs in an effort to protect the species from extinction. Since this oxbow was historically connected to the Yangtze River, its environmental and ecological conditions were believed to be similar to those of the Yangtze. Census data collected in November 2015 indicate that since 2010, the population has grown from 25 to over 60 individuals (Wang, 2015). Despite the importance of this population and the remaining individuals within their natural range, hematological and biochemical reference ranges for the YFP have yet to be established. Therefore, the objectives of this study were to establish normal hematological and biochemical analyte reference range intervals for the YFP within varying sex, maturational, and physiologic states and to determine if mean hematological analyte concentrations vary by geographic location.

## Materials and Methods

### Ethics Statement

The study strictly adhered to the Chinese law and ethical guidelines for wild animals. The ethical approvals (Y513161101, O813081101, O811031101, Y21Z041101, and Y45A131101) were obtained from the Ministry of Agriculture of the People’s Republic of China and the Research Ethics Committee of the Institute of Hydrobiology, Chinese Academy of Sciences, Wuhan, China.

### Animal Collection and Sampling

The “sound chase and net capture” method (Hua, 1987) was used for the YFP collection both in the TZO and PL. Detailed information about the animal chasing, handling, and blood sampling is summarized in the studies of Hao et al. (2009) and Nabi et al. (2017a,b, 2018a). The methodology during the chasing, handling, and timing for blood sampling was consistent for both populations. All the sampled animals from both populations were individually identified and tagged. Approximately 10 ml of the blood was drained randomly once at a single event aseptically from the main vein of the tail fluke using a disposable 10-ml syringe (Gemtier, G/Ø/L: 21/0.7/31 mm, 201502, Shanghai, China). Approximately 2 ml of the blood was transferred into heparinized tubes (Nihon, 161-8560, Tokyo, Japan) for complete blood profile and the remaining into a serum separator and EDTA Vacutainer^®^ tubes (BD Vacutainer, Becton Dickinson, Franklin Lakes, NJ, United States). The samples were centrifuged (Eppendorf AG, 22332, Hamburg, Germany) at 1,500 × *g* for 15 min. The obtained serum was transferred into cryotubes (Thermo Fisher Scientific, Pittsburgh, PA, United States) and stored in a liquid nitrogen kettle until analysis.

### Study Design

Animals were grouped based on total body length (Gao and Zhou, 1993) and sex as follows: juvenile male (JM; <138 cm), adult male (AM; ≥138 cm), juvenile female (JF; <130 cm), and adult females (AF; ≥131 cm). AFs were further classified as non-pregnant (NP) non-lactating, pregnant females (P), lactating (L), and pregnant plus lactating (P&L) based on ultrasonography (LOGIQ Book XP, New York, NY, United States) of the reproductive tract and presence of milk in the mammary glands. The information about sampling frequency both in TZO and PL is provided in Table 1.

**Table 1:** Number of samples collected from finless porpoise (FP) during capture and release health surveys conducted in Poyang Lake (PL) and Tian-E-Zhou Oxbow (TZO).

Site	Samples analyses	Survey periods	Juvenile^1^	NP female	Pregnant female	AF	AM	Total adults	Total FP/site
PL	Hematology	2009–2017	42	13	19	32	35	67	**109**
TZO	Hematology	2002–2015	28	11	12	23	58	81	**79**
		**Total FP/group**	**70**	**24**	**31**	**55**	**93**	**148**	**188**

PL	Serum Chemistry	2009–2017	41	0	24	24	32	56	**97**
TZO	Serum Chemistry	2002–2015	15	4	9	13	40	53	**68**
		**Total FP/group**	**56**	**4**	**33**	**37**	**72**	**109**	**165**

*^1^JFs < 130 cm in total length. JMs < 138 cm in total length. Within hematology data, Fisher’s exact test indicated that when data were combined across both locations, no differences were detected in sample distribution between age group (adults and juvenile) and sex (P = 1.0) or between age group and location (P = 0.06). However, significant differences were detected in numbers of AM and female samples between locations (P = 0.017). This difference did not exist (P = 0.17) when samples collected from only adult NP females and AMs compared between locations were examined. For serum chemistry data, overall no differences in frequency of sampling of adults and juvenile across each sex were detected (P = 0.727). However, when pregnant females were excluded, the distribution was different (P < 0.001). Finally, no differences were detected in the frequency of AMs and all AFs (P = 0.07) or AMs and NP (P = 0.134) females across locations.*

### Laboratory Analysis

Complete blood count; eosinophils, basophils, monocytes, lymphocytes, neutrophils, white blood cells (WBCs), red cell distribution width (RDW), platelet distribution width (PDW), platelet crit (PCT), mean platelet volume (MPV), platelets (PLT), mean corpuscular volume (MCV), mean corpuscular hemoglobin concentration (MCHC), mean corpuscular hemoglobin (MCH), hematocrit (HCT), hemoglobin (Hb), and red blood cells (RBCs) were analyzed using a hematology analyzer (Beckman-Coulter, DxH 800, Porto, Portugal) according to the manufacturer’s instruction.

Electrolytes [PO4^3−^, Ca^2+^, K^+^, Na^+^, Cl^−^, Mg^2+^, and Fe^2+^], enzymes [lactate dehydrogenase (LDH), creatine kinase (CK), amylase (AMS)], lipid profile [triglyceride (TG), total cholesterol (TC), low-density lipoprotein cholesterol (LDL-c), high-density lipoprotein cholesterol (HDL-c)], liver function parameters [total bile acid (TBA), indirect bilirubin (I-BILI), direct bilirubin (D-BILI), total bilirubin (T-BILI), gamma-glutamyl transferase (GGT), alkaline phosphatase (ALP), aspartate amino transferase (AST), alanine amino transferase (ALT)], and other biochemical parameters such as creatinine (Cr), uric acid (UA), carbon dioxide (CO_2_), blood urea nitrogen (BUN), glucose (GLU), globulin (GLB), Albumin (ALB), and total protein (TP) were measured using the automated clinical chemistry analyzer (Beckman-Coulter, AU5400, Porto, Portugal).

### Statistical Analysis

Based on sampling methodology and for the analysis, animals were considered to have been randomly selected during each collection period, and no animal was sampled more than once. All statistical analyses were performed using STATA (version 14, StataCorp LP, College Station, TX, United States). Briefly, outliers were identified as values greater than 3 SD above the mean for each stratified age by sex group. Total removed outliers for each variable were less than 0.61% (±0.003%). The equalities of sampling frequency between potential groups (age, group, and sex) were evaluated using Fisher’s exact test.

#### Reference Interval Calculations

Prior to calculations of reference intervals, the need to separate data into groups based on age and sex was evaluated as described by Schwacke et al. (2009). Briefly, pairwise intergroup distribution differences were determined between each age group (adult vs. juvenile), AMs vs. females and pregnant and NP females as follows. First, the pairwise intergroup distribution differences at the 2.5 and 97.5 percentiles were determined using the bootstrapped resampling technique (1,000 reps), and then the resultant 95% confidence interval (CI) of the distribution difference between groups was considered significant if it did not include zero. The *t*-test statistic was used to provide the estimated probabilities for this difference. Analytes which needed to be partitioned by sex, age group, and pregnancy were then established based in part on whether or not differences existed between the comparisons.

Once the need to partition the data by sex, age group, or pregnancy was established, the reference intervals were calculated based on the recommendations from the Clinical and Laboratory Standards Institute (Clinical and Laboratory Standards Institute [CLSI], 2008) using a non-parametric bootstrap-based procedure (Linnet, 2000; International Aids Vaccine Initiative, 2008). Using bootstrap resampling techniques (1,000 reps), the mean 2.5 and 97.5 percentiles from 1,000 resampling replicates of each group were determined along with the 90% bootstrapped CIs for each mean, respectively. As recommended by Linnet (2000), bootstrapped 90% CIs could only be determined for groups with samples from a minimum of 40 individuals. For analytes that had values which represented less than 40 individuals, only the range of these values was reported (Linnet, 2000; Schwacke et al., 2009).

Since analyte values were combined from two different locations, it was necessary to determine if this combination of data from both locations resulted in intervals which were not representative of values from both locations. We used the method described by Schwacke et al. (2009) for this determination. Briefly, the number of samples from each location that were outside the 2.5 and 97.5% reference was determined. This proportion (total outside upper or lower percentile/total samples collected from group) was then compared to the maximum predicted probability (0.025) that that sample would fall outside either the upper or lower value using a binomial probability test.

#### Effect of Location on Hematology Data

While differences in mean biochemistry data between locations have been recently reported (Nabi et al., 2018b), no such comparisons have been made for hematology data. For this analysis, hematological analytes were compared between locations using a restricted maximum likelihood (REML) mixed model with maturity (juvenile vs. adults) set as the random variable. By setting the age group as the random variable, we were able to determine if analyte clustering around the two age groups provided significant variance to the overall model (West et al., 2015). We chose to assume that clustering around age group had a significant effect due to previous multiple reports that age group provides a significant influence on the analyte values in other species (Venn-Watson et al., 2007; Schwacke et al., 2009; Norman et al., 2013; Nollens et al., 2019) and for biochemical values in YFP (Nabi et al., 2018b). In addition, since we were only interested in determining if age group had a significant effect on the means and not in producing predicted values for age group (while controlling for location and sex), this approach appeared most efficient. The statistical software (STATA) utilized automatically calculates the likelihood ratio test for the full model (which includes the random variable age group) versus a simple linear regression without the random variable. This provides evidence that the clustering of data around the means of each age group is significant (*P* < 0.05). In addition to the fixed variable location, we added sex (0 = female and 1 = male) and season (winter: December to February, spring: March to May, summer: June to August, fall: September to November) as covariates. All final mixed models were checked for normality using quantile plots of the standard residuals. If quantile–quantile (qnorm) plots of standardized residuals exhibited non-normal distribution, then data were transformed as predicted by the Shapiro–Wilk test until residuals were normalized. Finally, pairwise comparisons of the marginal means between and within fixed variables were made while applying the Sidak correction factor. Unless specified, data in tables and figures are presented as mean ± SEM and 95% CIs, and significance was set at *P* ≤ 0.05.

## Results

A combined maximum of 188 samples were collected from finless porpoise (FP) located at either PL or TZO from 2009 to 2017 and from 2002 to 2015, respectively. Sampling frequencies from each potential class are listed in Table 1. Within hematology data, Fisher’s exact test indicated that when data were combined across both locations, no differences were detected in sample distribution between age group (adults and juvenile) and sex (*P* = 1.0) or between age group and location (*P* = 0.06). However, significant differences were detected in numbers of AMs and females sampled between locations (*P* = 0.017). This difference did not exist (*P* = 0.17) when samples collected from only adult NP females and AM were compared between locations were examined. For serum chemistry data, overall, no differences in frequency of sampling of adults and juvenile across each sex was detected (*P* = 0.727). However, when pregnant females were excluded, the distribution was different (*P* < 0.001). Finally, no differences were detected in the frequency of AM and all AFs (*P* = 0.07) or AMs and NP females (*P* = 0.134) across locations (Table 1). However, since all but four females were pregnant in the biochemical data and pregnancy is a known influencer on biochemical analytes across species, and due to the small sample size from females (total sample size from all females being less than 40), we chose to partition data from NP and pregnant animals and report the range of analyte values as a starting point for future analyses. In addition, since the sampling frequency of pregnant females across locations was significantly different (*P* = 0.011), comparisons of analytes were only made with results combined across both locations between AM and pregnant females.

### Reference Interval Calculations

For partitioning, 29% of the juvenile samples were significantly different that those from adults, combined with evidence in other cetaceans of the importance of age group (Venn-Watson et al., 2007; Schwacke et al., 2009; Norman et al., 2013; Nollens et al., 2019) and for ease of reporting, references intervals for all analytes in juveniles were reported (Table 2). Within the hematology, there was a 13% significant difference between AM and AF (Tables 3, 4). Similarly, in adult males, 46% of the analytes were significantly different than pregnant females, and thus, values were also partitioned (Table 4). However, since only four NP females had data collected for biochemistry and pregnant females had a large degree of variation from AMs, these groups were partitioned separately for reference interval determination (Tables 3, 4).

**Table 2:** Reference interval limits (2.5th, 50th, and 97.5th percentiles) and the associated 90% bootstrap (1,000 reps) CIs for hematological and biochemical analytes determined for juvenile, as defined by total body length (male < 138 cm and female < 130 cm in length), FPs that were evaluated by capture and release programs conducted in PL and TZO from 2002 to 2017 (Nabi et al., 2018b).

Analyte	No. of porpoises	2.5% (90% CI)	50% (90% CI)	97.5% (90% CI)
**Hematology**				
HCT (%)^∗^	68	39 (36–41)	49 (47–50)	68 (59–78)
Hb (g/dl)	70	12.3 (10.2–14.4)	16.9 (16.5–17.3)	21.4 (18.8–24.0)
RBCs (×10^6^ cells/μl)	69	4.4 (4.1–4.6)	5.2 (5.1–5.3)	6.1 (5.7–6.4)
MCV (fl)	61	82 (76–87)	95 (94–96)	109 (106–111)
MCH (pg)	60	29 (28–30)	33 (32–33)	37 (36–38)
MCHC (g/dl)	60	30 (29–31)	34.5 (33.6–35.4)	42 (39–44)
RDW (%)	34	11.8 (11.2–12.4)	13.4 (13.0–13.7)	16.3 (14.9–17.7)
PLT (10^3^ cells/μl)	70	48 (45–52)	135 (127–143)	220 (200–240)
MPV (fl)	58	8.2 (6.1–10.2)	12.3 (11.7–12.9)	16.4 (13.6–9.3)
PDW (%)	61	14.5 (13.7–15.3)	16.6 (16.0–17.2)	56.9 (55.1–58.7)
WBCs (×10^3^ cells/μl)^∗^	68	3.94 (3.68–4.21)	7.60 (7.19–8.00)	13.09 (10.13–16.04)
Neutrophils (×10^3^ cells/μl)^∗^	36	2.66 (ND)	4.44 (3.96–4.92)	9.81 (ND)
Lymphocytes (×10^3^ cells/μl)^∗^	33	0.53 (ND)	2.65 (2.31–2.99)	4.83 (ND)
Mono (cells/μl)^∗^	36	14.4 (ND)	116 (101–131)	0.42 (ND)
Eosinophils (cells/μl)	35	2.3 (ND)	394 (112–665)	1.74 (1.29–2.19)
Basophils (cells/μl)^∗^	37	0 (ND)	12 (5–19)	0.07 (ND)
**Serum chemistry**				
GLU (mmol/l)	46	5.7 (5.5–5.9)	7.5 (7.1–7.9)	12.4 (11.2–13.6)
BUN (mmol/l)	56	6.9 (NA)	18.2 (17.3–19.1)	26.2 (24–28)
Cr (μmol/l)	55	33 (20–46)	78 (74–82)	120 (116 –124)
UA (μmol/l)	56	7.6 (NA)	47 (42–54)	229 (142–316)
TBAs (μmol/l)	56	1.0 (0.3–1.8)	4.8 (3.8–6.2)	18.5 (16.0–21.0)
T-BILI (μmol/l)	53	0.04 (NA)	3.2 (2.7–3.7)	9.1 (5.0–13.1)
D-BILI (μmol/l)	56	0 (NA)	1.0 (0.7–1.3)	2.9 (2.6–3.1)
I-BILI (μmol/l)	56	0.2 (NA)	1.7 (1.3–2.0)	9.1 (5.1–13.1)
Cholesterol (mmol/l)	51	0.8 (NA)	6.0 (5.6–6.3)	8.1 (7.7–8.4)
TG (mmol/l)^∗^	50	0.3 (0.2–0).5	0.9 (0.8–0.9)	3.2 (1.9–4.6)
HDL-c (mmol/l)	56	0.4 (NA)	2.8 (2.6–2.9)	3.7 (3.5–3.9)
LDL-c (mmol/l)	56	0.04 (NA)	0.2 (0.2–0.3)	3.6 (1.4–5.9)
HDL-c/LDL-c	56	0.6 (NA)	10.6 (8.1–11.8)	147 (13–280)
TP (g/l)^∗^	56	58 (56–61)	71 (70–73)	86 (83–88)
ALB (g/l)^∗^	56	20.2 (4.5–35.8)	42.3 (39.1–45.5)	65.9 (63.5–68.4)
GLB (g/l)	56	2.4 (NA)	28 (25–31)	50.6 (40.6–60.6)
ALB/GLB	55	0.5 (NA)	1.4 (1.2–1.7)	13 (11–15)
ALP (U/l)^∗^	54	102 (84–119)	240 (205–275)	855 (704–1006)
ALT (U/l)^∗^	56	7.4 (4.6–10.2)	33.5 (31.1–35.9)	75.9 (69.2–82.5)
AST (U/l)	56	93 (67–119)	211 (204–218)	435 (297–573)
AST/ALT^∗^	56	2.8 (1.9–3.7)	6.4 (5.9–6.8)	22.3 (14.5–30.2)
GGT (U/l)	52	20 (17–24)	35 (32–38)	79 (67–91)
CK (U/l)^∗^	48	14.6 (6.6–22.5)	163 (134–192)	468 (342–595)
LDH (U/l)^∗^	48	115 (102–128)	260 (227–292)	514 (425–521)
AMS (U/l)	47	1.0 (NA)	7 (6–8)	40 (24–42)
Calcium (mmol/l)^∗^	53	2.2 (2.2–2.3)	2.6 (2.59–2.64)	2.9 (2.8–2.9)
Phosphorus (mmol/l)^∗^	50	0.4 (0.2–0.6)	1.6 (1.5–1.8)	2.7 (2.5–2.9)
Sodium (mmol/l)	56	144 (141–148)	156 (154–157)	168 (160–175)
Chloride (mmol/l)	56	101 (100–102)	108 (106–109)	122 (116–129)
Potassium (mmol/l)	56	2.7 (2.0–3.5)	4.2 (4.0–4.5)	6.7 (5.5–7.9)
Mg (mmol/l)	56	0.59 (0.56–0.62)	0.78 (0.71–0.85)	1.3 (1.1–1.6)
CO_2_ (mmol/l)	45	14 (13–17)	24 (21–25)	36 (30–37)
Iron (μmol/l)	38	0.7 (NA)	31 (NA)	57 (NA)

*For sample sizes < 40, bootstrapped CIs could not be determined; therefore, only minimum, 50th percentile, and maximum analyte values are provided. ^∗^Represents analytes which are different from adults (AMs, NP females, and pregnant females), as determined by bootstrap t-test (1,000 reps, P < 0.05) of 95% CI. NA, not applicable; ND, not determined.*

**Table 3:** Reference interval limits for hematological and biochemical analytes for pregnant female, as determined by ultrasonographic examination, and NP AF (≥130 cm in total body length) FPs that were evaluated by capture and release programs conducted in PL and TZO from 2002 to 2017 (Nabi et al., 2018b).

Analyte	Adult NP female		Pregnant female	
	No. of porpoises	Range	No. of porpoises	Range
**Hematology**				
HCT (%)^a^	22	52 (40–61)^∗^	29	48 (32–60)
Hb (g/dl)^a^	22	17 (14–19)	31	16.0 (12.1–18.8)^∗^
RBCs (×10^6^ cells/μl)^a^	22	5.2 (4.2–6.1)	31	4.9 (3.4–6.1)
MCV (fL)	20	100 (87–109)	26	96 (89–111)
MCH (pg)	20	33 (32–34)	26	33 (29–39)
MCHC (g/dl)	20	32 (31–35)^∗^	26	33 (31–39)
RDW (%)	18	13 (12–16)	21	13 (12–16)
PLT (10^3^ cells/μl)	22	132 (55–217)	31	109 (37–186)
MPV (fl)	19	12 (10–16)	24	12 (10–14)
PDW (%)	21	17 (16–25)	26	17 (16–24)
WBCs (×10^3^ cells/μl)	22	5.86 (4.80–15.3)	31	6.04 (3.58–10.5)
Neutrophils (×10^3^ cells/μl)	15	4.33 (2.92–6.22)	19	3.97 (1.88–5.63)
Lymphocytes (×10^3^ cells/μl)	13	1.30 (0.73–1.93)	21	1.33 (0.84–3.48)
Monocytes (cells/μl)	15	97 (28–226)	21	72 (4–183)
Eosinophils (cells/μl)	15	515 (56–1426)	21	401 (75–1,795)
Basophils (cells/μl)	16	7 (0–37)	21	6 (0–15)
**Serum chemistry**				
GLU (mmol/l)	4	8.5 (7.8–8.7)	23	7.4 (5.07–10.9)
BUN (mmol/l)	4	19 (16–23)	33	20 (12–29)^∗^
Cr (μmol/l)	4	77 (68–98)	33	80 (46–129)
UA (μmol/l)	4	46 (26–78)	33	43 (10–393)
TBAs (μmol/l)	4	5 (4–6)	29	5 (1.2–19.8)
T-BILI (μmol/l)	4	2.8 (1.6–4.1)	32	3.5 (1–18.0)
D-BILI (μmol/l)	4	0.4 (0.1–0.6)	32	1.0 (0–3.9)
I-BILI (μmol/l)	4	2.5 (1.3–3.7)	27	1.5 (0.3–17.9)
Cholesterol (mmol/l)	4	8.8 (5.6–9.4)	30	5.4 (3.8–12.1)
TG (mmol/l)	4	0.7 (0.6–1.8)	30	1.2 (0.7–5.7)^∗^
HDL-c (mmol/l)	4	3.9 (2.7–4.1)	29	2.5 (1.7–3.3)^∗^
LDL-c (mmol/l)	4	0.5 (0.2–0.6)	29	0.3 (0.1–1.2)^∗^
HDL-c/LDL-c	4	8 (6–12)	29	11 (0–38)^∗^
TP (g/l)	4	71 (61–74)	33	77 (63–100)^∗^
ALB (g/l)	4	55 (52–58)	33	43 (35–66)
GLB (g/l)	4	16 (9–17)	29	36 (3–50)^∗^
ALB/GLB	4	4 (3–6)	29	1.2 (1–15)^∗^
ALP (U/l)	4	88 (83–97)	33	91 (38–574)
ALT (U/l)	4	52 (41–88)	33	35 (6–80)^∗^
AST (U/l)	4	245 (190–284)	33	182 (1–305)^∗^
AST/ALT	4	5 (3–6)	33	5 (0–23)
GGT (U/l)	4	30 (23–34)	33	36 (19–61)^∗^
CK (U/l)	4	148 (109–173)	33	102 (11–269)
LDH (U/l)	3	224 (221–227)	29	211 (71–429)
AMS (U/l)	4	6 (5–9)	28	6 (0–374)
Calcium (mmol/l)	4	2.6 (2.6–2.7)	33	2.5 (2.1–2.9)
Phosphorus (mmol/l)	4	1.5 (1.2–1.9)	29	1.5 (0.5–2.7)^∗^
Sodium (mmol/l)	4	154 (153–156)	33	157 (147–165)
Chloride (mmol/l)	4	107 (104–111)	33	109 (101–118)
Potassium (mmol/l)	4	5.0 (4.5–6.9)	33	4.3 (3.5–7.2)^∗^
Mg (mmol/l)	4	0.59 (0.5–0.6)	19	0.8 (0.7–1.2)^∗^
CO_2_ (mmol/l)	4	30 (27–37)	21	19 (8–31)^∗^
Iron (μmol/l)	4	30 (22–38)	20	32 (6–59)

*Since a maximum of 33 pregnant dolphins and 22 NP AFs were sampled, only 50th percentile, minimum, and maximum (range) analyte values could be provided. **^a^**Represents analytes which are different between pregnant and NP AFs as determined by bootstrap t-test (1,000 reps, P < 0.05) of 95% CI. **^∗^**Represents analytes which are different from AMs as determined by bootstrap t-test (1,000 reps, P < 0.05) of 95% CI. The small serum chemistry sample size from NP AFs prohibited comparisons against adult pregnant females and AMs.*

**Table 4:** Reference interval (2.5th, 50th, and 97.5th percentiles) and the associated 90% bootstrap (1,000 reps) CIs for hematological and biochemical analytes determined for AM (total body length ≥ 138 cm) FPs that were evaluated by capture and release programs conducted in PL and TZO from 2002 to 2017 (Nabi et al., 2018b).

Analyte	No. of porpoises	Lower limit (90% CI)	50th percentile (90% CI)	Upper limit (90% CI)
**Hematology**				
HCT (%)^∗^	83	37 (35–40)	47 (46–48)	61 (57–66)
Hb (g/dl)	90	14.1 (13.6–14.6)	16.5 (16.0–16.7)	19.8 (18.8–20.8)
RBCs (×10^6^ cells/μl)	89	4 (3.6–4.5)	5.0 (4.9–5.1)	6.4 (6.1–6.8)
MCV (fL)	75	82 (80–84)	94 (92–96)	117 (112–123)
MCH (pg)	81	29.1 (28.3–30.0)	32.4 (31.9–32.9)	36.5 (35.9–37.0)
MCHC (g/dl)	75	304 (301–306)	349 (338–360)	417 (405–429)
RDW (%)	33	12.1 (ND)	13.3 (13.1–13.5)	16.9 (ND)
PLT (10^3^ cells/μl)	81	199 (36–49)	130 (121–139)	199 (139–258)
MPV (fl)	68	9.6 (8.6–10.6)	12.1 (11.7–12.4)	15.5 (14.6–16.3)
PDW (%)	55	0.06 (0.01–0.09)	16.6 (16.2–17.0)	19.5 (18.7–20.4)
WBCs (×10^3^ cells/μl)^∗^	89	3.67 (3.38–3.97)	5.80 (5.53–6.07)	8.88 (8.69–9.16)
Neutrophils (×10^3^ cells/μl)^∗^	46	2.43 (2.25–2.61)	3.77 (3.43–4.11)	6.76 (6.27–7.26)
Lymphocytes (×10^3^ cells/μl)^∗^	49	0.49 (0.45–0.52)	1.26 (1.07–1.45)	3.49 (2.97–4.01)
Mono (×10^3^ cells/μl)^∗^	44	8.6 (3.1–14.0)	87 (74–100)	236 (188–285)
Eosinophils (×10^3^ cells/μl)	52	8.4 (NA)	334 (150–818)	1.79 (0.95–2.63)
Basophils (×10^3^ cells/μl)^∗^	43	0 (NA)	6.7 (5.0–8.4)	0.04 (0.02–0.05)
**Serum chemistry**				
GLU (mmol/l)	64	5.2 (3.3–7.1)	7.7 (7.4–8.0)	10.9 (9.6–12.2)
BUN (mmol/l)	72	6.5 (3.6–9.3)	17.5 (16.7–18.3)	10.9 (9.6–12.2)
Cr (μmol/l)	71	40 (33–47)	78 (74–83)	113 (103–123)
UA (μmol/l)	69	13 (9–16)	40 (37–44)	112 (88–135)
TBAs (μmol/l)	63	0.9 (0.4–1.4)	5.4 (4.3–6.5)	27 (17–36)
T-BILI (μmol/l)	69	0.5 (0.4–0.6)	3.0 (2.6–3.4)	9 (7–11)
D-BILI (μmol/l)	70	0 (NA)	0.5 (0.13–0.87)	4.4 (3.4–5.3)
I-BILI (μmol/l)	67	0.27 (0.2–0.4)	1.9 (1.6–2.2)	9.2 (6.8–11.7)
Cholesterol (mmol/l)	67	2.3 (1.1–3.6)	6.2 (5.8–6.5)	9.2 (8.1–10.4)
TG (mmol/l)^∗^	66	0.42 (0.35–0.50)	0.8 (0.7–0.9)	2.5 (1.6–3.4)
HDL-c (mmol/l)	62	0.8 (0.3–1.3)	2.9 (2.8–3.1)	3.8 (3.7–3.9)
LDL-c (mmol/l)	62	0.15 (0.1–0.2)	0.5 (0.3–0.6)	3.3 (2.6–3.9)
HDL-c/LDL-c	62	0.8 (0.2–1.3)	6.2 (4.1–8.3)	20 (17–24)
TP (g/l)^∗^	71	62 (61–64)	73 (72–74)	101 (86–116)
ALB (g/l)^∗^	71	36 (35–37)	45.6 (42.8–48.4)	68 (66–71)
GLB (g/l)	71	4 (1–6)	27.3 (25.5–29.1)	51 (48–55)
ALB/GLB	71	0.74 (0.62–0.86)	1.6 (1.5–1.7)	22.7 (4.2–41.2)
ALP (U/l)^∗^	70	47 (38–55)	129 (112–146)	349 (291–407)
ALT (U/l)^∗^	70	10 (5–15)	38 (36–41)	103 (67–138)
AST (U/l)	72	123 (101–143)	204 (197–211)	401 (336–466)
AST/ALT^∗^	72	2.1 (0.6–3.7)	5.2 (4.8–5.5)	13.5 (10.4–16.5)
GGT (U/l)	72	27.5 (25.3–29.6)	41 (38.1–43.9)	84 (73.8–94.1)
CK (U/l)^∗^	60	14 (NA)	105 (91–119)	311 (221–401)
LDH (U/l)^∗^	62	118 (89–147)	210 (185–235)	463 (333–593)
AMS (U/l)	62	1.6 (NA)	9 (7–11)	42 (21–63)
Calcium (mmol/l)^∗^	71	2.16 (2.14–2.19)	2.5 (2.5–2.6)	2.84 (2.74–2.94)
Phosphorus (mmol/l)^∗^	62	0.5 (0.3–0.6)	1.2 (1.1–1.3)	3.1 (2.2–4.0)
Sodium (mmol/l)	70	144 (142–147)	154 (153–155)	164 (161–168)
Chloride (mmol/l)	70	100 (100–101)	110 (109–111)	118 (115–122)
Potassium (mmol/l)	71	2.8 (2.6–3.1)	4.1 (3.9–4.2)	7.3 (3.2–11.4)
Mg (mmol/l)	52	0.6 (0.5–0.6)	0.71 (0.67–0.73)	1.1 (0.9–1.3)
CO_2_ (mmol/l)	56	12 (9–15)	24.5 (22.7–26.2)	32 (30–34)
Iron (μmol/l)	41	9 (3–16)	27 (24–30)	99 (44–154)

*For sample sizes < 40, only minimum and maximum analyte values are provided, and therefore, CI could not be determined. ^∗^Represents analytes which are different from adults (AMs, NP females, and pregnant females), as determined by bootstrap t-test (1,000 reps, P < 0.05) of 95% CI. NA, not applicable; ND, not determined.*

### Reference Intervals Based on Location

Analysis of numbers of out-of-range samples from each analyte from the two locations indicates that only magnesium concentrations in AMs located at TZO had more values which were significantly less (5/19 = 0.26, *p* = 0.0001) than the predicted number.

### Differences in Mean Hematological Analyte Concentrations Between Locations

When controlling for sex and season, a total of 88% of the values were significantly different between locations (Table 5). The covariates sex and season had 31 and 69% significant difference, respectively, in mean distributions between locations. Finally, the random variable age group was a significant source of variation in 25% of the analytes. Significant increases in the blood cells (HCT, Hb, PLT, MCH, MCHC, MCV, and PDW) were observed in the animals located at TZO. However, the blood cells (MPV and RDW) and inflammatory cells (WBC, lymphocyte, monocyte, eosinophil, and basophil) were statistically significantly higher in the PL YFPs. The blood parameters (HCT, MCV, MCHC, MPV, and monocyte) showed significant variation with sex and with age in PLT, WBC, lymphocyte, and basophil. Season was a strong influencer, and all the parameters except RDW, MPV, WBC, neutrophil, and monocyte showed statistically significant variation.

**Table 5:** Comparison of marginal mean (95% CI) hematology values in YFP between location, with the covariates gender^1^ and season (W, winter; S, spring; F, fall). ^2^ Effects of age category (J, juvenile; A, adult) was evaluated by assigning it as the random variable.^3^ Predicted 95% CIs for each location while controlling for sex, age group, and season are listed below.

	Marginal mean (95% CI)		*P*-values			
Analyte	PL	TZO	Location	Sex	Age group	Season
HCT	48 (46–49)	50 (49–52)	0.003	0.02	0.11	<0.0001, S & F < W
Hb (g/dl)	164 (160–168)	169 (165–172)	0.048	0.49	0.16	<0.0001, S < W
RBCs (×10^6^ cells/μl)	5.2 (5.1–5.3)	5.1 (5.0–5.2)	0.24	0.66	0.16	<0.0001 S < W & F
MCV (fL)	93 (92–94)	99 (98–100)	<0.0001	0.0007	1.0	<0.0001, S & F < W
MCH (pg)	32.1 (31.7–32.5)	33.1 (32.7–33.4)	0.0007	0.54	1	0.0001, W & F < S
MCHC (g/dl)	346 (341–352)	337 (333–342)	0.03	0.002	1	<0.0001, W < F < S
RDW (%)	13.7 (13.4–13.9)	12.8 (12.3–13.3)	0.005	0.37	1	0.52
PLT (10^3^ cells/μl)	117 (101–133)	139 (123–156)	0.0002	0.5	0.01	<0.0001, S < W & F
MPV (fl)	13.3 (13.0–13.6)	11.5 (11.3–11.7)	<0.0001	0.01	0.48	0.59
PDW (%)	17 (14–20)	30 (27–34)	<0.0001	0.22	1.0	<0.0001, F < S < W
						
WBCs (×10^3^ cells/μl)	7.12 (6.26–8.08)	5.96 (5.25–6.78)	<0.0001	0.54	<0.0001	0.18
Neutrophils (×10^3^ cells/μl)	4.38 (3.84–4.99)	4.17 (3.71–4.68)	0.53	0.79	0.072	0.12
Lymphocytes (×10^3^ cells/μl)	2.59 (1.63–4.12)	1.34 (0.86–2.09)	0.0002	0.08	<0.0001	0.02 S < W & F
Mono (cells/μl)	108 (77–154)	67 (49–90)	0.03	0.049	0.17	0.24
Eosinophils (cells/μl)	455 (279–742)	193 (130–288)	0.007	0.37	0.2	0.0003, F < W & S
Basophils (cells/μl)	17 (11–25)	8 (5–12)	0.0007	0.68	0.006	<0.0001
Analytes affected (%)			88 (14/16)	31 (5/16)	25 (4/16)	69 (11/16) W & S < F

*The P-values for each of the fixed variables and the random variable (age group) are listed. Since season was composed of three periods, post hoc comparison Sidak comparisons (P < 0.05) were performed to determine intraseasonal differences. ^1^Due to sample size and for this analysis, AFs were combined without regard for pregnancy status. ^2^Only one sample was collected in the summer; therefore, this analyte was removed from the analysis. ^3^For this mixed model maximum likelihood regression analysis, age group was assigned as the random variable to account for clustering of values around age group. The significant effect on analyte variance due to the random variable, age group, was tested using the likelihood ratio test and *P*-values reported herein.*

## Discussion

In this study, reference intervals were made for 49 clinicopathological variables which were determined in 188 samples collected from 188 YFPs over a period of more than 17 years. These animals were located in two different geographical areas, TZO, a seminatural reserve, and PL, a portion of the native habitat of the YFP located in the middle of the Yangtze River. Results of our YFP reference interval delineation were similar to the reference intervals developed in other cetaceans, and mammals in general, in that strong evidence was provided which indicates that analytes should be grouped according to age (body length) and sex, and in females, pregnant versus NP animals (Venn-Watson et al., 2007; Schwacke et al., 2009; Norman et al., 2012, 2013; Nollens et al., 2019).

While differences in mean concentrations for biochemical analytes within each animal grouping between TZO and PL have been previously noted (Nabi et al., 2017a, 2018b), when we evaluated the need to partition reference ranges by location, we found that only one analyte, magnesium, within AMs at TZO had significantly more animals than would be predicted with values which were less than calculated reference intervals. In a previous study in YFP (Nabi et al., 2018b), mean magnesium concentrations were also found to be significantly lower in animals located at TZO. However, mean changes were also detected in all other electrolytes; thus, the isolated change observed in Mg^+2^ is difficult to assess clinically, but it appears to reflect the large difference in its serum concentration distribution in animals between these two locations. Despite this one exception, the fact that the frequency of animals which fell outside the reference range for all other analytes was as expected indicates that a need to partition the results by location was not evident. Generally, therefore, our reported reference intervals, which combined results from animals in both locations, should prove satisfactory for the detection of pathology within individual animals at either location.

However, the lack of data collected from adult NP females and the evidence in other cetaceans which supports the concept for partitioning based on pregnancy within AFs (Harewood et al., 2000; Harvey et al., 2005; Robeck and Nollens, 2013) indicate a need to collect more data from NP AFs and that the analyte ranges provided in this study should only be considered as a starting point for evaluating the clinical health of other adult YFP females.

### Hematological Partitioning

While no evidence was found which indicated that hematological reference intervals required partitioning based on location, the HCT and all WBC analytes except eosinophils significantly varied by age and were, thus, partitioned. In several terrestrial (monkeys, rats, goats, donkeys, and dogs) and aquatic mammals (bottlenose dolphin, elephant seal, harbor seal, steller sea lions, and manatees), variation in the blood and biochemical parameters with respect to age and sex has been reported (Bossart et al., 2001; Venn-Watson et al., 2007; Gerlinsky et al., 2018). Only HCT and MCHC were different between sex (AM versus adult NP female), and this may have been a reflection of the small samples size of NP AFs (*n* < 22). When AMs were compared to all AFs (pregnant and NP females combined), HCT and all WBC indices except eosinophils were significantly different. The detection of inflammatory cell differences when combining female groups may simply have been a result of doubling the sampling size of females and, therefore, enabling the detection of differences between AMs and AFs. In some AF cetaceans, including YFPs (unpublished work), both the estrous cycle and pregnancy have been documented to affect the WBC counts (Venn-Watson et al., 2007; Robeck and Nollens, 2013). Continued collection of hematological data from both pregnant and NP females combined with ultrasound determination of gestational stage or follicular activity may eventually allow us to develop more sensitive reference ranges which are partitioned based on variations in hematological analytes during each physiologic state (pregnant, NP, and estrous cycle) found in AF YFP.

### Electrolyte Partitioning

As with hematological analytes, the distribution of serum concentrations of Ca^+2^ and PO^−4^ was significantly affected by age, and serum PO^−4^, K^+^, Mg^+2^, and CO_2_ were affected by sex. In other cetaceans, age significantly affect electrolytes (Kasamatsu et al., 2012; Gerlinsky et al., 2018). In addition, it has been postulated that higher levels of Ca^+2^ and PO^−4^ could correspond to skeletal growth (Reif et al., 2004) and that concentrations which are outside the upper reference interval limit may suggest dehydration (Trumble and Castellini, 2002). Like bottlenose dolphin (Kasamatsu et al., 2012), sex-related variation in the serum Ca^+2^ and PO^−4^ concentration was detected.

### Hepatic Partitioning

The hepatic enzymes (ALP, ALT, and AST/ALT) showed a significant variation across age groups, and therefore, they were partitioned by age. The ALP concentration has been shown to decrease with age in harbor porpoises (*Pbocoena pbocoena*), Atlantic bottlenose dolphin (*Tursiops truncatus*), and killer whales (Nollens et al., 2019). In cetaceans, ALP is associated with rapid bone development and, therefore, can be used as an indicator of physical maturity in YFPs (Andersen, 1968; Fair et al., 2006; Venn-Watson et al., 2007). Like YFPs, serum ALT also varies significantly with age and sex in the northern elephant (*Mirounga angustirostris*), harp (*Phoca groenlandica*), and hooded seals (*Cystophora cristata*) (Boily et al., 2006; Yochem et al., 2008). Furthermore, diet can also affect serum ALT level in cetaceans (Greig et al., 2010). The levels of GGT also differ between AMs and females like bottlenose dolphin (Venn-Watson et al., 2007). Unlike terrestrial mammals, AST and GGT are also found in the muscles, kidneys, and other organs of aquatic mammals and are therefore not solely related to liver diseases (Medway and Geraci, 1986). However, ALT appears to be liver specific in pilot whales (*Globicephala* spp.), Pacific white-sided dolphins (*Lagenorhynchus obliquidens*), and bottlenose dolphins (Bossart, 1984; Bossart et al., 1990, 1991) and, therefore, can be used for screening liver diseases.

### Lipid Profile Partitioning

In our study, lipid profiles had significantly different distributions with both age and sex. The TG had differences across the age and sex groups. The distribution of other parameters like HDL-c, LDL-c, and HDL-c/LDL-c also varied significantly with sex. The blubber thickness of *Eubalaena australis* calves increases during the first months of suckling while the calves of *E. glacialis* in the last months of suckling were as thick as juvenile whales due to the provision of positive energy balance by the mother’s milk to provide a high percentage of their body mass to blubber (Miller et al., 2011). As the percentage of blubber increases in calves, the level of polyunsaturated fatty acids increases, possibly for thermoregulation and buoyancy purposes. During the juvenile stage, the level of monounsaturated fatty acids is higher, which may be in response to rapid growth, restricted diving, and energy depletion (Stephen et al., 2010). Besides age (Goldstein et al., 2006), diet, reproductive status, and season also affect lipid composition and blubber thickness (Terasawa et al., 2002; Castellini et al., 2009). Similarly, sexual dimorphism in the cholesterol and TG levels has been reported in the bottlenose dolphin (Terasawa et al., 2002; Goldstein et al., 2006). The sexual dimorphism in lipid profile could be the result of sex-specific network of hormone actions in combination with direct or indirect modulators (insulin and adipokin) involved in lipid metabolism (Wang et al., 2011). Higher levels of cholesterol have been reported in captive pregnant bottlenose dolphin and killer whales compared to NP females, respectively (Terasawa and Kitamura, 2005; Robeck and Nollens, 2013). However, no such difference for pregnant vs. NP AFs has been reported in free-ranging bottlenose dolphin (Goldstein et al., 2006).

### Other Biochemical Parameter Partitioning

The variations of LDH and CK concentrations were significantly different between age groups, with a reduction in concentrations occurring with increasing age in the YFPs. A similar trend has been reported in beluga whales (*Delphinapterus leucas*) and steller sea lions (*Eumetopias jubatus*) (Tsai et al., 2016; Gerlinsky et al., 2018) and could possibly be linked to behavior or physiologic adaptation connected to the rut (Tryland et al., 2006). Furthermore, younger YFPs compared to adults may have more chances of skeletal damage during handling, causing elevation in CK level (Goldstein et al., 2006). Similarly, the variation observed in TP and ALB levels between the juveniles and adults could be related to variation in the diet (Medway and Geraci, 1986; Yochem et al., 2008). To cope with the higher energy demand during pregnancy, we observed significant variations in the BUN, TP, GLB, and ALB/GLB between pregnant females and AMs. During pregnancy, both the synthesis and catabolism of protein increase because of the higher energy demand for developing fetus. As a result of higher protein catabolism, the level of BUN also increases (Durak and Altinek, 2006; Omidi et al., 2015).

### Marginal Mean Comparisons of Hematological Analytes Between Locations Within Sex and Age Groups

Despite no difference in the numbers of dolphins outside of the reference range intervals in the two locations, we did observe a significantly higher mean concentration of hematological indices (HCT, Hb, PLT, MCH, MCHC, MCV, and PDW) in TZO vs. PL. These differences reinforce similar results, across a smaller sample size, in several hematological analytes, between mean concentrations found in animals residing in these two diverse locations (Nabi et al., 2017a). Many reasons including diseases, nutrition, and parasitic infestation can cause variation in the blood cell concentrations (Bossart et al., 2001; Castellini et al., 2010; Shiozaki and Amano, 2017). Similarly, the inflammatory cells (WBC, lymphocyte, monocyte, eosinophil, and basophil) were significantly higher in the PL vs. TZO as observed in the two studies by Nabi et al. (2017a,b). Variation in the inflammatory cells with respect to geographical location suggests variation in the level of stress, bacterial, viral and fungal infections, chronic infections, and parasitic infestations (Andersen, 1966; Medway and Geraci, 1986; Asper et al., 1990; Koopman et al., 1999). The age-related variations in the blood parameters (WBC, lymphocyte, eosinophil, and basophil) found in our study have also been reported in the harbor seal (*Phoca vitulina*) and small sample size of YFPs (Greig et al., 2010; Nabi et al., 2017a). Similarly, sex and seasonal variation first observed for YFP in this study have also been reported in other cetaceans (St Aubin et al., 2001; Boily et al., 2006; Trumble et al., 2006; Hall et al., 2007; Tsai et al., 2016; Nabi et al., 2017a).

By combining data from both locations, we were able compile a robust sample size which enabled us to partition data by age (length), AM, and AF. However, the consistent finding of mean hematological (this paper) and biochemical (Nabi et al., 2018b) analyte concentrations which vary by site provides evidence that future efforts should be directed toward collecting enough animals to allow for site-specific reference intervals. This is a goal that may or may not be feasible given the limited number of total animals within the remaining population. Until or if this goal has been achieved, the data reported within provide the first set of reference intervals for this critically endangered population and guidelines for the initial evaluation of individuals during health assessments.

## Data Availability

The raw data supporting the conclusions of this manuscript will be made available by the authors, without undue reservation, to any qualified researcher.

## Ethics Statement

The study strictly adhered to the Chinese law and ethical guidelines for wild animals. The ethical approval was obtained from the Ministry of Agriculture of the People’s Republic of China and the Research Ethic Committee of Institute of Hydrobiology, Chinese Academy of Sciences, Wuhan, China.

## Author Contributions

GN conceived the study and drafted the manuscript. TR analyzed the data and critically reviewed the manuscript. YH and DW collected the data, supervised and supported the study. All authors read and approved the final version of the manuscript.

## Conflict of Interest Statement

TR was employed by SeaWorld Parks and Entertainment, Orlando, United States. The remaining authors declare that the research was conducted in the absence of any commercial or financial relationships that could be construed as a potential conflict of interest.
